# Efficacy of an eHealth self-management program in reducing irritable bowel syndrome symptom severity: a randomized controlled trial

**DOI:** 10.1038/s41598-023-50293-z

**Published:** 2024-01-03

**Authors:** Jun Tayama, Toyohiro Hamaguchi, Kohei Koizumi, Ryodai Yamamura, Ryo Okubo, Jun-ichiro Kawahara, Kenji Inoue, Atsushi Takeoka, Shin Fukudo

**Affiliations:** 1 Faculty of Human Sciences, Waseda University, 2-579-15 Mikajima, Tokorozawa, Saitama 359-1192 Japan; 2 Department of Occupational Therapy, School of Health and Social Services, Saitama Prefectural University, 820, Sannomiya, Koshigaya, Saitama 343-8540 Japan; 3 Division of Biomedical Oncology, Institute for Genetic Medicine, Hokkaido University, Kita 15, Nishi 7, Kita-ku, Sapporo, Hokkaido 060-0815 Japan; 4 Department of Psychiatry and Neurology, National Hospital Organization Obihiro Hospital, 16, Kita 2, Nishi 18, Obihiro, Hokkaido 080-0048 Japan; 5 Department of Psychology, Hokkaido University, Kita 10, Nishi 7, Kita-Ku, Sapporo, Hokkaido 060-0815 Japan; 6 Center for Student Success Research and Practice, Osaka University, 1-2, Yamadaoka, Suita, Osaka 565-0871 Japan; 7 Health Center, Nagasaki University, 1-14, Bunkyo, Nagasaki, Nagasaki 852-8521 Japan; 8 Department of Behavioral Medicine, Tohoku University Graduate School of Medicine, 2-1, Seiryo-Machi, Aoba-ku, Sendai, Miyagi 980-8575 Japan

**Keywords:** Psychology, Patient education

## Abstract

This study aimed to verify whether an eHealth-based self-management program can reduce irritable bowel syndrome (IBS) symptom severity. An open-label simple randomized controlled trial was conducted that compared an intervention group (n = 21) participating in an eHealth self-management program, which involved studying IBS-related information from an established self-help guide followed by in-built quizzes, with a treatment-as-usual group (n = 19) that, except for pharmacotherapy, had no treatment restrictions. Participants were female Japanese university students. The eHealth group received unlimited access to the self-management program for 8 weeks on computers and mobile devices. The primary outcome, participants’ severity of IBS symptoms assessed using the IBS-severity index (IBS-SI), and the secondary outcomes of participants’ quality of life, gut bacteria, and electroencephalography alpha and beta power percentages were measured at baseline and 8 weeks. A significant difference was found in the net change in IBS-SI scores between the eHealth and treatment-as-usual groups, and the former had significantly lower IBS-SI scores following the 8-week intervention than at baseline. Moreover, there was a significant difference in the net change in phylum Cyanobacteria between the eHealth and treatment-as-usual groups. Thus, the eHealth-based self-management program successfully reduced the severity of IBS symptoms.

## Introduction

Irritable bowel syndrome (IBS) is a functional gastrointestinal disorder characterized by marked abnormality in brain–gut interaction in the absence of major organic abnormality^1^. Its main pathophysiological features include dysmotility of the lower gastrointestinal tract, visceral hypersensitivity, and psychological abnormalities^1,2^. IBS is highly prevalent worldwide, with adult prevalence rates of 10.1% and 4.1% according to the Rome III and IV criteria, respectively^3^. IBS is also associated with impaired daily functioning and a pronounced decline in quality of life (QoL), including interference with daily activities, health-related anxiety, and food avoidance^4^. Furthermore, the economic impact of having IBS is 1.1–6.0 times greater than that for non-IBS controls^5^.

The clinical practice guidelines for IBS recommend non-pharmacological therapies along with pharmacotherapy^6,7^. In both human^1,2^ and animal studies^8^, gastrointestinal symptoms of IBS or IBS-like gastrointestinal function were exacerbated by worsening psychiatric symptoms and improved by psychological recovery. Recently, cognitive-behavioral therapy has been shown to contribute to the improvement of IBS symptoms^9^. Other non-pharmacological therapies such as exercise therapy^10^ are also effective. With the recent advancements in understanding the relationship between intestinal microbiota and IBS symptoms, dietary therapies such as low FODMAP (fermentable oligosaccharides, disaccharides, and monosaccharides and polyols) diets have been attracting attention^11^.

Self-management has traditionally been important in controlling chronic disease symptoms^12,13^. Non-pharmacological treatments of IBS often incorporate self-management methods, wherein patients actively control their own symptoms. Self-management of IBS can ameliorate disease and economic burdens^14^. For example, the low FODMAP diet controls IBS symptoms and increases microbiota diversity by helping patients manage their food intake^11^.

Self-management of IBS using a self-help guidebook^15,16^ requires significant patient effort^14–16^. However, in a randomized controlled trial using the self-help guidebook with 420 patients with IBS in the United Kingdom, the intervention group had 60% fewer visits to primary care, less severe IBS symptoms, and 40% lower annual cost per patient than the control group, one year after the intervention^14^. A prospective observational study of 71 IBS patients in Germany using a self-help guidebook reported a significant increase in QoL six months after the intervention^16^. The framework of the original self-help guidebook includes three elements: involving patients in the development of information, changing access arrangements to health services, and promoting a patient-centered approach to care^17^. The self-help guidebook adapted in this study is designed to allow students to cover its educational content within two weeks, and enables them to self-check what they have learned through a checklist^15^. For our eHealth program, we modified the six chapters of this guidebook: “Personal experiences of IBS,” “Understanding IBS,” “What you can do to help yourself,” “More ways to manage your IBS,” “Medical treatments,” and “Summary and sources of information”^15,16^.

The present study introduces eHealth, a web-based practice that assists healthcare providers in ambulatory care, for IBS patients and evaluates its potential to enhance their self-management. The application of eHealth for IBS treatment and follow-up can alleviate symptoms, optimize patient compliance, improve QoL, and reduce the economic burden^18,19^. A previous study on 34 patients with IBS found comparable symptom reduction with eHealth-based probiotic treatments and a low FODMAP diet^18^. Regarding the IBS self-help guidebook adapted for this study^14–16^, its content is yet to be validated in the eHealth format.

This study's primary objective was to verify the hypothesis that an eHealth-based self-management program can reduce the severity of IBS symptoms.

## Methods

### Study design

This study was designed as an open-label simple randomized controlled trial with the intervention group receiving a self-management program through eHealth and a treatment-as-usual (TAU) group. This study was registered on 25/11/2020 at https://center6.umin.ac.jp/cgi-open-bin/ctr/ctr_view.cgi?recptno=R000047461 (UMIN Clinical Trials Registry, UMIN000042552).

### Participants

Participants were 40 symptomatic IBS patients meeting the Rome IV criteria and enrolled as university students in Japan. The diagnosis of IBS was made by a physician. Studies have shown that women and younger people are at a higher risk of IBS^3^, based on which we set the inclusion criterion as Japanese women aged 18–36 years. The exclusion criteria were having previously received pharmacotherapy for IBS, any preexisting psychiatric disorders, and other organic gastrointestinal diseases. All patients provided written informed consent to participate in this study. The study protocol was approved by the Ethics Committee of Saitama Prefectural University (Registration Number: 20048) and was conducted in accordance with the ethical standards laid down in the 1964 Declaration of Helsinki and its later amendments. Compliance with the study protocol was verified by the access logs of the eHealth system.

### The eHealth program

The five chapters in our program also included content from previous studies (Table 1)^15,16^. The eHealth program for IBS was designed for use on computers and mobile devices, allowing participants to download and store content locally for easy access and learning. Each chapter comprised text in an e-book format and narrated video for increased accessibility^20^. The web server for the eHealth program consisted of two primary components: Moodle and CHiLO Book. Built entirely on open-source software and cloud hosted, CHiLO Book^20^ plays a central role as a video delivery system and is embedded within Moodle. Its user interface adopts an e-book format, displaying scripts beneath the video, allowing users to read while watching. Upon completing a chapter in the eHealth program using CHiLO Book, users gained access to a quiz function in Moodle. The content was available for viewing for 8 weeks. The goal for participants was to study each chapter at least once and complete the quiz at the end. Assessments were conducted before the start of the eHealth intervention (baseline) and at the end (at 8 weeks).

**Table 1:** Elements included from each chapter of the eHealth program.

	Section	Contents/elements	Number of quizzes Quiz keywords
1. Understanding IBS	Introduction	What is irritable bowel syndrome?/medical tests and more serious problems	9 IBS symptoms Symptoms complicated with IBS Prevalent period of IBS Causes of IBS Foods that trigger symptoms Diagnosis and determination of IBS Stool abnormalities Gas symptoms of IBS IBS and sugar intake
	Causes of IBS	(Theoretical content) Diet/change in living environment/imbalance of intestinal bacteria/muscle contractions of large intestine/intestinal sensation/psychological stress/relationship with hormones	
	How the digestive system works	Gastrointestinal tract/small intestine/large intestine/peristalsis/role of intestinal wall and nerves/defecation/gas production	
	Diet and the digestive system	Dietary habits/dietary modification/exclusionary diet/lactose intolerance/fructose malabsorption/sorbitol malabsorption/celiac disease/fiber intake	
2. What you can do to help yourself	Dietary management	Modern diet/food compatibility/exclusionary diet/dairy products/processed foods/try to consume calcium/fruits, vegetables, artificial sweeteners/wheat products/drinks/abdominal bloating/intestinal bacteria	10 Unhealthy dietary content Wheat products and digestive symptoms Coping with lactose intolerance Nutrients in dairy products Harmful effects of coffee and alcohol Foods that produce gas Benefits of exercise Relaxation through breathing techniques Stress management Control of food intake
	Exercise	Benefits of exercise/tips on exercising	
	Managing psychosocial stress	What is stress/how to cope with stress	
	Relaxation	Effects of relaxation/relaxation techniques/muscle relaxation and relaxation/breathing and relaxation/jaw relaxation	
3. Additional ways to manage your IBS	Non-medical methods	Non-medical methods/getting treatment/active methods/talking to someone	6 Types of non-medical treatments Reflexology and massage Yoga Benefits of social support Chinese medicine and natural foods Harmful effects of laxative use
	Prescription-free remedies	Therapeutic medication/constipation aids/pain relievers/other products	
	Things to remember	Cost/how to use the pharmacy	
4. Medical treatments	Medications that require a prescription	Medications for constipation/treatment with antidepressants/types of antidepressants	4 Drug therapy for IBS Laxatives utilized in Japan Effects of antidepressants Relationship between pharmacotherapy and non-pharmacotherapy
	Other treatments	Cognitive-behavioral therapy/psychotherapy/hypnotherapy/surgery and IBS	
5. Useful information for self-management	Useful information for self-management	Overcoming IBS/general tips/exploring symptoms/medications/problems with sexual interactions/information on the internet	4 Recommended fluid intake Formation of defecation habits The harmful effects of too much dietary fiber Relationship between abdominal muscle exercise and intestinal digestion
	Current research	Purpose of medical research/drug research/relationship to daily life/new treatments	

*IBS* irritable bowel syndrome.

### Treatment as usual

For the TAU group, the eHealth program was not accessible; however, assessments similar to those of the intervention group were conducted at the same time points (baseline, 8 weeks). The TAU group did not receive any pharmacotherapy or non-pharmacotherapy from their healthcare providers. However, there were no restrictions on their daily non-pharmacologic self-management, such as exercise and diet therapy.

### Randomization

To minimize selection bias and ensure the impartial assignment of participants to the experimental and control groups, we employed a randomization procedure. We used computer-generated random numbers or a randomization table to achieve this, and the allocation sequence was concealed from the researchers to maintain integrity. To prevent any potential investigator bias, we conducted a single-blind study. All data collection and analysis procedures were conducted by a data analyst who was unaware of the group assignments. Following an open-label design, all participants were informed which group they were assigned to.

### Outcomes

The primary outcome measured was the total score of the IBS-severity index (IBS-SI, IBS-symptom severity scale, IBS-SSS)^21,22^ at 8 weeks after the intervention. The IBS-SI is utilized to evaluate the severity of gastrointestinal symptoms. It comprises five items, and the total score ranges from 0 to 500. Patients with IBS have significantly higher IBS-SI scores than healthy subjects^21,22^. Secondary outcomes were the total score of the IBS-QoL measure^4,23^, electroencephalography (EEG) alpha and beta power percentages, compositions of the gut microbiota (at the phylum, order, class, family, and genus levels), and α-diversity indices of the gut microbiota. The IBS-QoL comprises 34 items with 5-point Likert scales (0 to 4). Higher values indicate better QoL after converting the raw score on the IBS-QoL into a range of 0 to 100 points. In addition, intake based on the low FODMAP diet was qualitatively evaluated. Specifically, an original questionnaire was developed, and responses were sought regarding the amount of low FODMAP foods consumed in the past month from seven food groups: breads and cereals, vegetables, fruit, milk and dairy, protein, nuts and seeds, and beverages. Patients with IBS are known to have lower QoL than healthy individuals^4,23^, as well as lower baseline EEG alpha power and higher baseline beta power than controls^24^. In addition, two systematic reviews have shown that patients with IBS have abnormal gut microbiota compared to healthy individuals^25,26^. Moreover, adopting a FODMAP diet is known to contribute to the transformation of gut bacteria in IBS patients^11^. Therefore, these outcomes are considered suitable for the interventions conducted in this study.

### Sample size

The sample size was determined based on a previous study that investigated improvement in the IBS-SI from a 3-week non-pharmacological treatment for patients with IBS^11^. The study found a reduction in the mean IBS-SI score following the intervention (mean ± SD treatment group; IBS-SI score 208.0 ± 74.8 and TAU group 290.0 ± 106.0). From this, we estimated that ≥ 17 individuals per group were required for a difference in the IBS-SI score ≥ 82.0 (SD = 31.2) with an α level of 0.05 (two-tailed) and 80% power.

### EEG recording and quantitative EEG analysis

EEG recordings were performed at baseline and 8 weeks after the intervention using Ag/AgCl electrodes placed at 11 sites according to the international 10–20 system^27^. EEG data were recorded under the eyes-closed condition using a Polymate AP6000 system (TEAC Co., Ltd., Tokyo, Japan) for 15 min before the start of the eHealth program. The reference electrode was placed on the left earlobe and the impedance set to < 10 kΩ. Outcome measures were the power spectra of the alpha and beta bands.

Two-minute segments of EEG data were collected during the 15-min recording period. EEG data were obtained at the same time points in each group. Data were analyzed through fast Fourier transform using appropriate software (MP Viewer Pro; Miyuki Giken, Tokyo, Japan; See Supplemental Table S1)^24^.

### Bacterial DNA extraction and microbiome analysis

Bacterial DNA was extracted from feces samples using a nucleic acid extraction system, PI-1200 (Kurabo, Osaka, Japan). Each library was prepared according to the Illumina 16S Metagenomic Sequencing Library Preparation Guide with a primer set, 27Fmod/338R, targeting the V1–V2 region of 16S rRNA genes. The 251-bp paired-end sequencing of the amplicons was performed on a MiSeq system (Illumina, CA, USA) using a MiSeq Reagent v2 500 cycle kit. All steps from the trimming of the paired-end reads FASTQ files obtained via 16S rRNA amplicon sequencing, to the gut microbiota analysis were performed using QIIME 2^28^. First, the raw sequence results were demultiplexed and the DADA2 algorithm was used to identify microbial operational taxonomic units. We then classified the operational taxonomic units into five taxonomic rank categories (phylum, order, class, family, and genus) using the SILVA 132 reference database at 99% similarity. The Shannon index (H’) and Simpson index (1-D), which measure α-diversity, were calculated using the following equations at the genus level: H′ = − Σ*p*_*i*_ln*p*_*i*_ and D = Σp_i_^2^, where *p*_*i*_ is the relative abundance (%) of genus *i* in the community. Changes in the Shannon and Simpson indices in the eHealth and TAU groups at baseline and 8 weeks after intervention were analyzed using paired t-tests and analysis of covariance (ANCOVA).

### Statistical analysis

Data were expressed as mean ± SD. ANCOVA was used to assess the differences between mean scores, 95% confidence intervals, and p values for each outcome. Covariates for the ANCOVA were the continuous variables of age and body mass index (BMI), the discrete variable of IBS subtype (IBS with diarrhea [IBS-D], IBS with constipation [IBS-C], mixed IBS [IBS-M], and unsubtyped IBS [IBS-U]) and the continuous baseline scores for each outcome. ANCOVA was conducted after confirming its assumptions including normality of data, regression line parallelism, and regression significance. A two-tailed test was used with the α level set at 0.05%. The p value was calculated using Bonferroni correction. In accordance with a previous report^22^, we defined an IBS-SI score of 175 or higher as moderate to severe IBS and calculated the percentage of IBS for each time course to test the difference in proportions. Regarding intestinal bacteria, we applied the linear discriminant analysis effect size (LEfSe)^29^ with default settings to determine the features of the gut microbiota (at the phylum, order, class, family, and genus levels) that likely explain the differences in each group (eHealth vs. TAU).

### Ethical considerations

All patients provided written informed consent to participate in this study. The study protocol was approved by the Ethics Committee of Saitama Prefectural University (no. 20048).

## Results

### Demographic data

**Figure 1:**
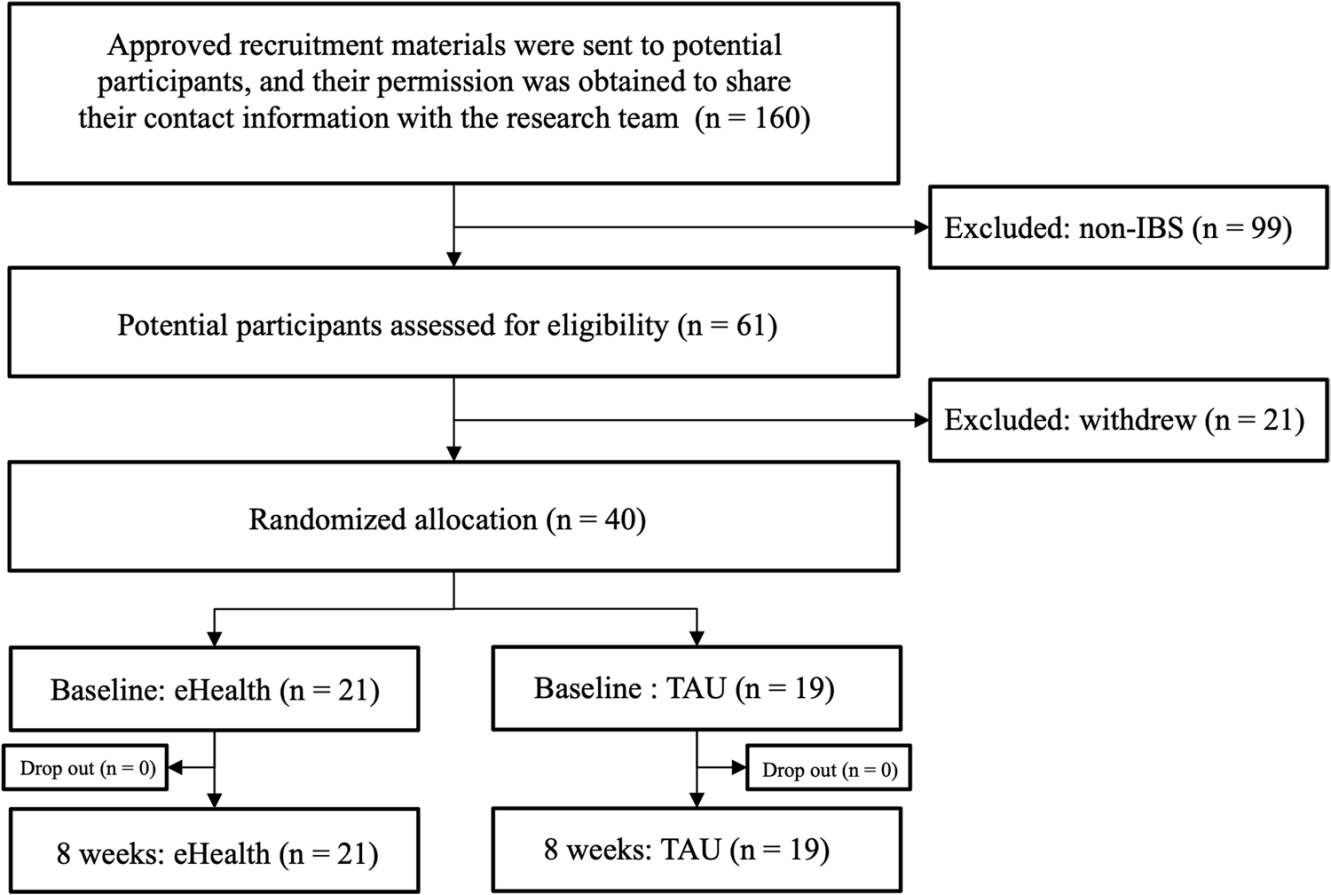
Recruitment, eligibility, and randomization of participants. Of the 160 initial recruits in this study, 99 were non-IBS at screening and 21 later withdrew. Of the 40 IBS symptomatic individuals, 19 were randomly assigned to the eHealth group for the 8-week eHealth intervention and 21 to the TAU group. *IBS* irritable bowel syndrome, *TAU* treatment as usual.

Prospective participants (n = 160) received a recruitment packet approved by the ethics committee and consented to share their contact information with the research team. Of the 160 approached, 99 were non-IBS at screening and excluded, resulting in 61 potential participants assessed for eligibility. Of the 61, 21 withdrew from participation. Finally, of the 40 remaining patients, 21 were randomly assigned to the eHealth group and 19 to the TAU group. All 40 participants (100%) successfully completed the randomized controlled trial without any dropouts. All 21 participants (100%) in the eHealth group accessed the content of all five chapters present in the eHealth program and completed each of the chapter quizzes at least once (Fig. 1).

Table 2 presents the baseline demographic data. Patients were well matched for age, BMI, and IBS subtype between the groups. The total scores of the IBS-SI and IBS-QoL at baseline, two of the outcomes measured in this study, were also well matched. No significant differences were observed between the two groups.

**Table 2:** Baseline characteristics of participants.

	eHealth	TAU	p value
	n = 21, 51%	n = 19, 49%	
Age, mean (SD), years	20.4 (1.0)	21.8 (3.9)	0.55
Sex, n (%)^†^			
Female	21 (100)	19 (100)	1.00
BMI, kg/m^2^	21.0 (1.5)	21.1 (2.3)	0.94
IBS type, n (%)^†^			
IBS-D	6 (29)	6 (32)	1.00
IBS-C	5 (24)	2 (11)	0.23
IBS-M	7 (33)	5 (26)	0.49
IBS-U	3 (14)	6 (32)	0.26
Baseline symptom severity, mean (SD)			
IBS-SI total score	200.7 (88.1)	198.8 (78.5)	0.94
Moderate and severe IBS, n (%)^†^	12 (63)	14 (67)	0.33
Baseline QoL, mean (SD)			
IBS-QoL total score	75.4 (16.9)	79.2 (12.6)	0.43

Moderate and severe IBS, IBS-SI ≧ 175.

*TAU* treatment as usual, *IBS-SI* irritable bowel syndrome-severity index, *IBS-QoL* irritable bowel syndrome-quality of life measure, *BMI* body mass index, *IBS-C* IBS with constipation, *IBS-D* IBS with diarrhea, *IBS-M* mixed type IBS, *IBS-U* unsubtyped IBS.

^†^Fisher’s exact analysis was used.

### Primary outcome measure: IBS-SI

**Figure 2:**
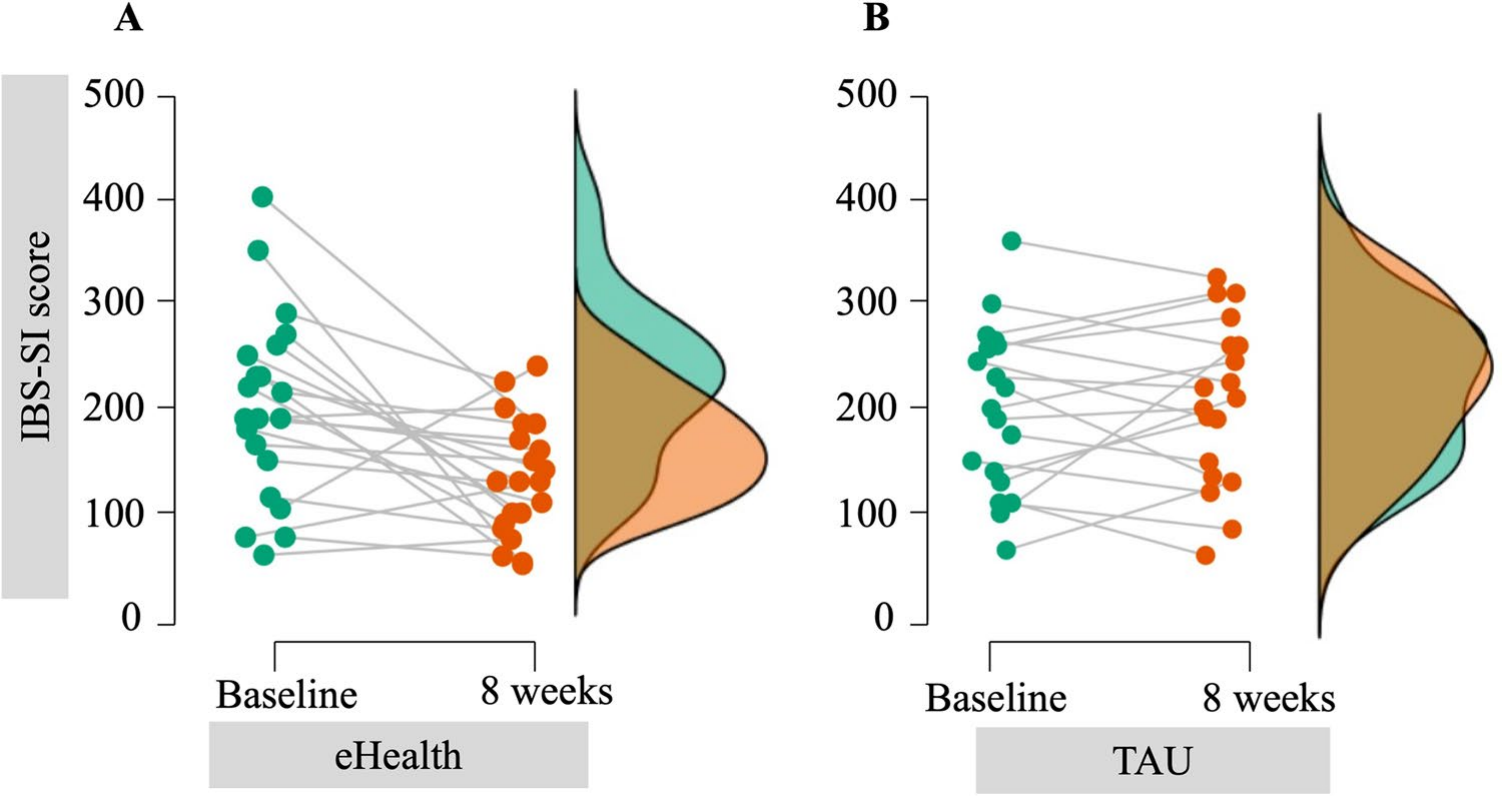
Time course plots of the changes in the total score of the IBS-SI in the eHealth and TAU groups. (**A**) Plots of the eHealth group. (**B**) Plots of the TAU group. The vertical axis represents the total score of the IBS-SI. Colored clouds in the right panel show the total score of the IBS-SI distributions according to the survey periods (green = baseline; orange = 8 weeks). *IBS* irritable bowel syndrome, *IBS-SI* irritable bowel syndrome-severity index, *TAU* treatment as usual.

Table 3 summarizes the data at baseline and 8 weeks for the primary outcome, the IBS-SI score. There was a significant difference in the net change in the IBS-SI scores between the eHealth and TAU groups (− 50.1; 95% CI − 87.6 to − 12.6; p = 0.010). Furthermore, the eHealth group had significantly lower IBS-SI scores after the 8 weeks of treatment than at baseline (t = − 3.2, p < 0.01). Figure 2 shows a time course plot of the change in the total IBS-SI scores in the eHealth and TAU groups.

**Table 3:** IBS-SI and IBS-QoL scores at baseline and 8 weeks.

	n	Baseline Mean (SD)	8 weeks Mean (SD)	Paired *t*-test		Net change (95% CI)	ANCOVA
				*t*	p value		p value
IBS-SI							
eHealth	21	200.7 (88.1)	131.9 (55.3)*	− 3.2	< 0.01	− 50.1 (− 87.6, − 12.6)	0.010
TAU	19	198.8 (78.5)	205.9 (77.5)	0.5	< 0.61		
IBS-QoL							
eHealth	21	75.4 (16.9)	88.1 (10.8)*	3.9	< 0.01	6.9 (0.5, 13.2)	0.034
TAU	19	79.2 (12.6)	77·5 (13.8)	− 0.5	< 0.59		

ANCOVA adjusted for age (continuous variable), BMI (continuous variable), IBS subtype (IBS-C, IBS-D, IBS-M, IBS-U), and baseline IBS-SI or baseline IBS-QoL score (continuous variable).

*TAU* treatment as usual, *IBS-SI* irritable bowel syndrome-severity index, *IBS-QoL* irritable bowel syndrome-quality of life.

*Statistically significant (p < 0.01) difference between eHealth and TAU in Student's t-test.

### Secondary outcome measure: IBS-QoL

Table 3 summarizes the data for the secondary outcome, IBS-QoL scores, at baseline and 8 weeks. There was a significant difference in the net change in the IBS-QoL scores between the eHealth and TAU groups (6.9; 95% CI 0.5–13.2; p = 0.034). Furthermore, the eHealth group had significantly higher IBS-QoL scores following the 8 weeks of treatment than at baseline (t = 3.9, p < 0.01).

### Secondary outcome measure: percentage of moderate and severe IBS

**Figure 3:**
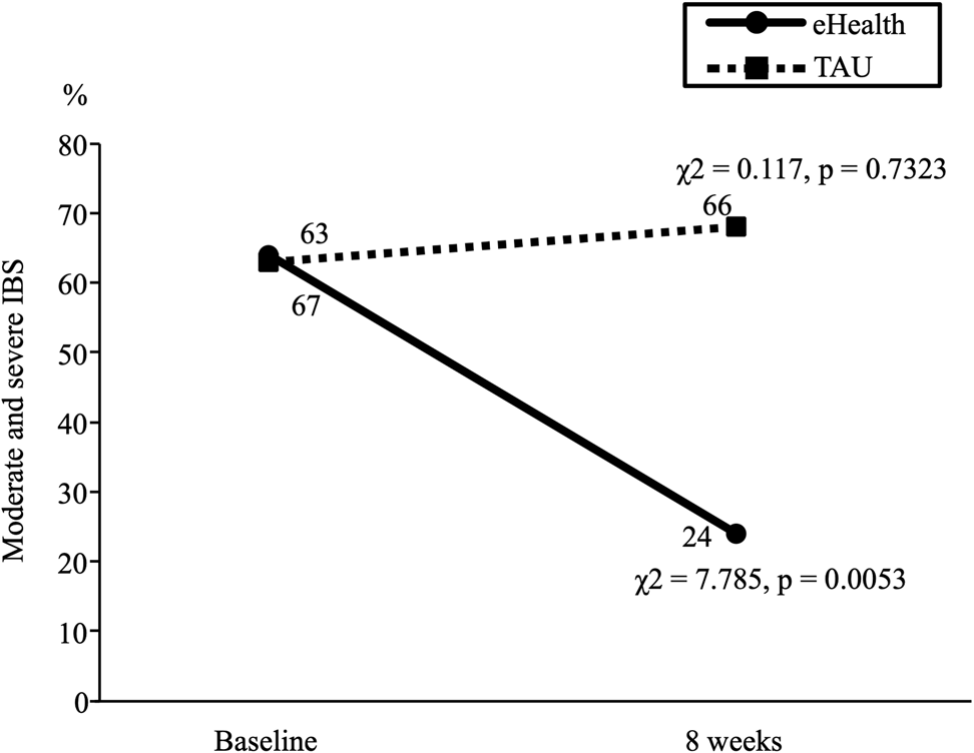
Changes in the percentage of moderate and severe IBS in both the groups. The solid line with circled markers is the eHealth group. The dashed line with square markers is the TAU group. The percentage of patients with moderate and severe IBS in the time course did not change significantly in the TAU group. The percentage change in the eHealth group over time was significantly different. *IBS* irritable bowel syndrome, *TAU* treatment as usual.

Figure 3 shows the time course changes in the percentage of moderate and severe IBS (IBS-SI ≧ 175) in both the groups. The percentage of patients with moderate and severe IBS in the time course did not change significantly in the TAU group (63% (n = 12) to 68% (n = 13), χ^2^ = 0.117, p = 0.7323). The percentage change in the eHealth group over time was significantly different (67% [n = 14] to 24% [n = 5], χ^2^ = 7.785, p = 0.0053).

### Secondary outcome measure: alpha and beta power percentages

Supplementary Table S1 shows the EEG alpha and beta power percentages measured in each brain region. There was no significant difference in the net change between the eHealth and TAU groups.

### Secondary outcome measure: phylum-level compositions and α-diversity indices of the gut microbiota

Table 4 shows the phylum-level compositions and α-diversity indices of the gut microbiota. In the phylum-level compositions, there was a significant difference in the net change in phylum Cyanobacteria between the eHealth and TAU groups (− 0.01; 95% CI − 0.02 to − 0.01; p = 0.001). Otherwise, there was no significant difference in the net change between the eHealth and TAU groups in any of the other phylum-level compositions. At baseline, four out of the 21 participants in the eHealth group had phylum Cyanobacteria, but after 8 weeks, three of these four had 0% occupancy of phylum Cyanobacteria. On the other hand, in the TAU group, one of the 19 participants had phylum Cyanobacteria at baseline and only that participant still had cyanobacteria after the intervention. Furthermore, there was no significant difference in the net change between the eHealth and TAU groups in the α-diversity indices (Shannon and Simpson indices) of the gut microbiota.

**Table 4:** Phylum-level compositions and α-diversity indices of gut microbiota at baseline and 8 weeks.

	n	Baseline Mean (SD)	8 weeks Mean (SD)	Paired *t*-test		Net change (95% CI)	ANCOVA
				*t*	p value		p value
Phylum (%)							
Actinobacteria							
eHealth	21	3.13 (1.96)	3.21 (2.43)	0.21	< 0.21	0.67 (− 0.64, 2.01)	0.298
TAU	19	4.95 (3.31)	3.64 (2.62)	− 2.09	< 0.06		
Bacteroidetes							
eHealth	21	37.44 (5.85)	39.69 (8.52)	1.42	< 0.17	− 0.97 (− 5.86, 3.92)	0.690
TAU	19	36.08 (8.62)	38.82 (6.59)	1.63	< 0.12		
Firmicutes							
eHealth	21	55.37 (5.96)	52.86 (9.18)	1.62	< 0.12	− 2.21 (− 7.06, 2.65)	0.361
TAU	19	55.73 (9.51)	54.70 (8.01)	− 0.62	< 0.55		
Proteobacteria							
eHealth	21	2.57 (1.28)	2.45 (0.9)	− 0.43	< 0.67	0.42 (− 0.15, 1.00)	0.144
TAU	19	2.56 (1.37)	1.91 (1.08)	− 2.54	< 0.03		
Acidobacteria							
eHealth	21	0.00 (0.00)	0.01 (0.01)	1.00	< 0.33	–	–
TAU	19	0.00 (0.00)	0.00 (0.00)	–	–		
Cyanobacteria							
eHealth	21	0.04 (0.18)	0.02 (0.07)	− 1.06	< 0.30	− 0.01 (− 0.02, − 0.01)	0.001
TAU	19	0.02 (0.09)	0.01 (0.03)	− 1.00	< 0.33		
Epsilonbacteraeota							
eHealth	21	0.01 (0.01)	0.01 (0.01)	0.19	< 0.85	0.01 (− 0.01, 0.01)	0.321
TAU	19	0.01 (0.01)	0.01 (0.01)	− 0.68	< 0.51		
Fusobacteria							
eHealth	21	1.33 (5.12)	1.67 (6.65)	1.00	< 0.33	− 0.11 (− 0.39, 0.16)	0.405
TAU	19	0.60 (2.13)	0.81 (2.75)	1.41	< 0.17		
Lentisphaerae							
eHealth	21	0.01 (0.01)	0.00 (0.00)	− 1.00	< 0.33	–	–
TAU	19	0.00 (0.00)	0.00 (0.00)	–	–		
Omnitrophicaeota							
eHealth	21	0.01 (0.01)	0.00 (0.00)	− 1.00	< 0.33	–	–
TAU	19	0.00 (0.00)	0.00 (0.00)	–	–		
Patescibacteria							
eHealth	21	0.01 (0.01)	0.01 (0.01)	− 1.08	< 0.29	− 0.01 (− 0.01, 0.01)	0.243
TAU	19	0.01 (0.01)	0.01 (0.01)	0.57	< 0.58		
Spirochaetes							
eHealth	21	0.00 (0.00)	0.01 (0.01)	1.00	< 0.33	–	–
TAU	19	0.01 (0.03)	0.00 (0.00)	− 1.00	< 0.33		
Synergistetes							
eHealth	21	0.00 (0.00)	0.00 (0.00)	–	–	–	–
TAU	19	0.00 (0.00)	0.00 (0.00)	–	–		
Tenericutes							
eHealth	21	0.00 (0.00)	0.01 (0.02)	1.00	< 0.33	–	–
TAU	19	0.00 (0.00)	0.00 (0.00)	–	–		
α-diversity indices							
Shannon index							
eHealth	21	7.02 (0.41)	7.01 (0.51)	− 0.12	< 0.91	0.03 (− 0.22, 0.27)	0.830
TAU	19	7.00 (0.55)	7.07 (0.45)	0.62	< 0.54		
Simpson index							
eHealth	21	0.98 (0.01)	0.98 (0.01)	− 0.27	< 0.79	0.00 (0.00, 0.01)	0.870
TAU	19	0.98 (0.01)	0.98 (0.01)	0.68	< 0.51		

ANCOVA adjusted for age (continuous variables), BMI (continuous variables), IBS subtype (IBS-C, IBS-D, IBS-M, IBS-U), and baseline phylum-level compositions and α-diversity indices (continuous variables). The p value was calculated using Bonferroni correction.

*TAU* treatment as usual.

### Secondary outcome measure: LEfSe to determine the features of the gut microbiota

No differences in the gut microbiota were found between the eHealth and TAU groups at each timepoint, before and after the intervention, by LEfSe (data not shown).

### Qualitative assessment of low FODMAP food intake status

We asked participants the quantity of low FODMAP foods they consumed in the past month from seven food groups: breads and cereals, vegetables, fruit, milk and dairy, protein, nuts and seeds, and beverages. At baseline, there were no group differences in the percentage of the seven low FODMAP food groups consumed. However, at week 8, only low FODMAP milk and dairy products had a higher percentage intake in the eHealth group than in the TAU group (24% [n = 5] vs. 0% [n = 0], p = 0.0230). Regarding the change in time course, there was no significant change in the TAU group for all seven low FODMAP food groups. In the eHealth group, the percentage of those eating low FODMAP foods increased from 71% (n = 15) to 95% (n = 20) in the nuts and seeds group (χ^2^ = 4.286, p = 0.0384). Among the eHealth participants, although there was an increase from 71% (n = 15) to 86% (n = 18) in the low FODMAP breads and cereals group (χ^2^ = 1.273, p = 0.2593), this was not significant. Similarly, in the low FODMAP milk and dairy group, intake increased from 10% (n = 2) to 24% (n = 5; χ^2^ = 1.543, p = 0.2142), and in the low FODMAP protein group (χ^2^ = 2.100, p = 0.1473), it increased from 90% (n = 19) to 100% (n = 21).

## Discussion

The results of this study suggest that the eHealth program reduces IBS symptom severity and improves QoL owing to two main reasons: extensive food-related content and a wide range of non-food content in various categories.

First, the eHealth program included several sections that provided detailed information on food-related aspects that may affect IBS symptoms, such as “diet and the digestive system” in Chapter 1 and “dietary management” in Chapter 2, which included details of the low FODMAP diet. Existing IBS eHealth programs focus primarily on dietary therapy that involves regulating FODMAP and probiotics^18,19^, and have already been shown to improve IBS symptoms. The secondary outcome of this study, low FODMAP food intake status, was normalized by the eHealth program. In our study, the eHealth program significantly increased the percentage of people consuming low FODMAP nuts and seeds. Furthermore, at the 8-week point, the percentage of individuals who consumed low FODMAP milk and dairy products was higher in the eHealth group compared to the TAU group. The second chapter of the eHealth program included content on “coping with lactose intolerance” and “nutrients in dairy products,” suggesting that learning about these food-related topics may have led to an optimized diet and subsequently reduced the severity of IBS.

Second, the variety of content in the eHealth program may have helped reduce the IBS-SI and improved QoL. The original self-help guidebook contained evidence-based information and techniques associated with IBS symptom improvement^15,16^, and similar content was included in our eHealth version. The first chapter covered “Understanding IBS,” and the subsequent chapters provided extensive knowledge on pharmaceutical and non-pharmaceutical interventions for IBS. Additionally, the eHealth program offered a range of content on non-dietary measures, such as exercise^10^, cognitive-behavioral therapy^9^, and relaxation^30^, known to help reduce IBS symptoms.

Furthermore, the prevalence of cyanobacteria, a gate-level intestinal bacterium, was reduced by the eHealth program in this study. Cyanobacteria produce a toxin called cyanotoxin, which causes diarrhea and other digestive symptoms when consumed with drinking water^31,32^. The reduction in cyanobacteria seen in this study may have contributed to a reduction in the severity of IBS symptoms, and likely occurred for two reasons. First, the contribution of a low FODMAP diet. In a study of pediatric IBS, children susceptible to fructan, a high FODMAP, had higher cyanobacterial levels^33^. A study examining the impact of a low FODMAP diet on IBS symptoms found an improvement in intestinal health alongside an improvement in IBS symptoms^11^. The improvement in food intake by learning about food in the eHealth program in our study may have reduced cyanobacterial occupancy. Second, a high proportion of the participants had diarrheal IBS, which may have indirectly contributed to the decrease in cyanobacteria. In an animal study, diarrhea model mice had significantly higher levels of cyanobacteria than normal mice^34^, suggesting that cyanobacteria exacerbate the symptoms of diarrheal IBS. In this study, participants had more diarrheal IBS (12 IBS-D and 12 IBS-M, 60%) in both the eHealth and the TAU groups.

The resting EEGs of IBS patients have been found to have lower alpha power and higher beta power than that of normal populations^24^; however, the EEG was not normalized by the eHealth program in this study. Changes in intestinal bacteria are known to affect brain function. The alteration of gut microbiota leading to EEG changes has already been established in both animal^35^ and human^36^ research. There are two possible reasons why the EEGs did not normalize in this eHealth program. First, being a non-pharmacological treatment, the impact of the eHealth program on brain function may not have been captured by the baseline and 8-week EEG comparisons. Pharmacological therapy in IBS patients tends to have a rapid impact on the brain^37^. In contrast, non-pharmacological therapy using the eHealth program employed in this study may have the potential for delayed effects on brain function. Second, although the eHealth program had a direct effect on the gastrointestinal tract, the indirect effect on the brain via improvement of gastrointestinal tract symptoms may not have been observable. Pharmacological treatment results in the normalization of gastrointestinal symptoms, leading to desensitization of the ascending signals from the gut to the brain, which in turn improves brain function^37^ and psychological states^38^. However, in the context of this research's eHealth program, the limited occurrence of these effects suggests the possibility that EEG changes did not take place.

Despite the insightful results, this study has some limitations. First, the study did not adjust for food intake as a confounding factor. The assessment of food intake was solely qualitative, despite the known effects of dietary changes on IBS symptoms^18,19^. In the future, reliable quantitative evaluations are needed to assess the impact of this study’s eHealth program on changes in food intake. Second, this study does not adjust for other lifestyle factors such as sleep^39^ and exercise^10^, which also affect IBS symptoms, although to a lesser extent. Third, the findings of the effectiveness of the eHealth program established in this study cannot be generalized. Further, owing to the study design, it was not possible to demonstrate effectiveness by severity or age group. A larger sample is required in future, to further clarify the effectiveness of the eHealth program in greater detail.

## Conclusion

The eHealth-based self-management program designed in this study reduced the severity of IBS symptoms. In terms of secondary outcomes, QoL was improved. In the phylum-level composition of gut microbiota, the eHealth program reduced the proportion of cyanobacterial phylum. However, it did not normalize the EEG alpha and beta power percentages measured in each brain region.

### Supplementary Information

Supplementary Table 1.

## Data Availability

All data relevant to the study are included in this article or uploaded as supplementary information S1.
