# Supplementary material for: Efficacy of an eHealth self-management program in reducing irritable bowel syndrome symptom severity: a randomized controlled trial
Source: Sci Rep. 2024 Jan 3;14:4. doi: 10.1038/s41598-023-50293-z (PMC10764726; doi:10.1038/s41598-023-50293-z)
Supplement: Supplementary file 1 — Supplementary Table 1. [file 41598_2023_50293_MOESM1_ESM.docx]

| Supplementary Table 1. EEG alpha and beta power percentages at baseline and 8 weeks | | | | | | | | | |
| --- | --- | --- | --- | --- | --- | --- | --- | --- | --- |
|  | | | n | Baseline Mean (S.D.) | 8 weeks Mean (S.D.) | Paired *t*-test | | Net change (95% CI) | ANCOVA |
|  |  |  |  |  |  | *t* | p value |  | p value |
| Alpha (%) | | |  |  |  |  |  |  |  |
|  | F3 | |  |  |  |  |  |  |  |
|  |  | eHealth | 21 | 27.6 (14.9) | 18.2 (10.3) | -2.74 | < 0.01 | -2.7 (-9.2, 3.7) | 0.391 |
|  |  | TAU | 19 | 17.4 (10.7) | 17.3 (9.2) | -0.05 | < 0.52 |  |  |
|  | Fz | |  |  |  |  |  |  |  |
|  |  | eHealth | 21 | 28.8 (15.0) | 18.9 (11.4) | -2.70 | < 0.01 | -3.7 (-10.8, 3.4) | 0.297 |
|  |  | TAU | 19 | 18.2 (10.7) | 18.9 (9.9) | 0.26 | < 0.40 |  |  |
|  | F4 | |  |  |  |  |  |  |  |
|  |  | eHealth | 21 | 27.7 (14.9) | 16.1 (11.4) | -3.10 | < 0.01 | -5.1 (-11.9, 1.7) | 0.138 |
|  |  | TAU | 19 | 16.2 (10.0) | 17.4 (9.2) | -0.52 | < 0.61 |  |  |
|  | C3 | |  |  |  |  |  |  |  |
|  |  | eHealth | 21 | 27.3 (15.2) | 22.2 (11.9) | -1.27 | < 0.22 | -1.0 (-8.4, 6.4) | 0.791 |
|  |  | TAU | 19 | 20.2 (10.9) | 20.7 (11.9) | -0.18 | < 0.86 |  |  |
|  | Cz | |  |  |  |  |  |  |  |
|  |  | eHealth | 21 | 31.7 (14.4) | 22.6 (11.4) | -2.55 | < 0.02 | -2.9 (-10.4, 4.5) | 0.430 |
|  |  | TAU | 19 | 21.0 (13.2) | 22.3 (12.4) | 0.38 | < 0.70 |  |  |
|  | C4 | |  |  |  |  |  |  |  |
|  |  | eHealth | 21 | 30.9 (15.7) | 22.6 (11.2) | -2.11 | < 0.05 | -2.3 (-9.4, 4.7) | 0.506 |
|  |  | TAU | 19 | 21.0 (12.0) | 21.7 (11.4) | 0.26 | < 0.80 |  |  |
|  | P3 | |  |  |  |  |  |  |  |
|  |  | eHealth | 21 | 33.8 (15.0) | 26.6 (13.1) | -2.01 | < 0.06 | -3.8 (-13.1, 5.4) | 0.403 |
|  |  | TAU | 19 | 25.2 (13.4) | 27.8 (14.2) | 0.67 | < 0.51 |  |  |
|  | Pz | |  |  |  |  |  |  |  |
|  |  | eHealth | 21 | 36.9 (15.9) | 27.3 (14.8) | -2.31 | < 0.03 | -4.7 (-14.6, 5.2) | 0.336 |
|  |  | TAU | 19 | 25.7 (13.8) | 29.0 (14.8) | 0.88 | < 0.39 |  |  |
|  | P4 | |  |  |  |  |  |  |  |
|  |  | eHealth | 21 | 26.8 (14.3) | 34.5 (19.5) | -1.69 | < 0.11 | 3.5 (-5.9, 13.0) | 0.450 |
|  |  | TAU | 19 | 27.5 (14.5) | 27.6 (14.4) | -0.01 | < 0.99 |  |  |
|  | O1 | |  |  |  |  |  |  |  |
|  |  | eHealth | 21 | 45.5 (19.5) | 34.3 (16.7) | -2.39 | < 0.03 | -7.4 (-17.6, 2.9) | 0.152 |
|  |  | TAU | 19 | 36.4 (19.1) | 37.7 (18.6) | 0.35 | < 0.73 |  |  |
|  | O2 | |  |  |  |  |  |  |  |
|  |  | eHealth | 21 | 47.4 (20.6) | 32.5 (18.4) | -2.64 | < 0.02 | -9.3 (-21.5, 2.9) | 0.130 |
|  |  | TAU | 19 | 41.8 (19.0) | 40.6 (20.7) | -0.24 | < 0.81 |  |  |
| Beta (%) | | |  |  |  |  |  |  |  |
|  | F3 | |  |  |  |  |  |  |  |
|  |  | eHealth | 21 | 5.8 (2.0) | 6.5 (3.7) | 0.95 | < 0.35 | 0.9 (-1.6, 3.4) | 0.461 |
|  |  | TAU | 19 | 7.1 (6.1) | 6.6 (3.7) | -0.45 | < 0.65 |  |  |
|  | Fz | |  |  |  |  |  |  |  |
|  |  | eHealth | 21 | 5.7 (1.9) | 6.3 (3.0) | 0.83 | < 0.41 | 1.0 (-1.7, 3.6) | 0.467 |
|  |  | TAU | 19 | 6.3 (3.3) | 6.8 (4.1) | 0.62 | < 0.54 |  |  |
|  | F4 | |  |  |  |  |  |  |  |
|  |  | eHealth | 21 | 5.9 (2.2) | 5.5 (3.0) | -0.54 | < 0.59 | -1.2 (-3.5, 1.2) | 0.335 |
|  |  | TAU | 19 | 7.4 (8.5) | 7.1 (4.4) | -0.15 | < 0.89 |  |  |
|  | C3 | |  |  |  |  |  |  |  |
|  |  | eHealth | 21 | 6.2 (2.6) | 7.3 (3.2) | 1.42 | < 0.17 | 1.1 (-1.6, 3.8) | 0.417 |
|  |  | TAU | 19 | 7.5 (3.8) | 7.7 (5.1) | 0.15 | < 0.88 |  |  |
|  | Cz | |  |  |  |  |  |  |  |
|  |  | eHealth | 21 | 6.9 (2.1) | 7.2 (2.8) | 0.39 | < 0.70 | -0.2 (-2.7, 2.3) | 0.868 |
|  |  | TAU | 19 | 6.5 (3.3) | 7.7 (4.7) | 0.94 | < 0.36 |  |  |
|  | C4 | |  |  |  |  |  |  |  |
|  |  | eHealth | 21 | 6.8 (2.6) | 7.2 (2.9) | 0.54 | < 0.59 | 0.1 (-2.5, 3.0) | 0.946 |
|  |  | TAU | 19 | 7.7 (4.0) | 8.1 (5.3) | 0.40 | < 0.70 |  |  |
|  | P3 | |  |  |  |  |  |  |  |
|  |  | eHealth | 21 | 7.3 (2.7) | 7.2 (3.2) | -0.24 | < 0.82 | -0.6 (-3.2, 2.0) | 0.633 |
|  |  | TAU | 19 | 8.0 (3.9) | 8.6 (5.0) | 0.57 | < 0.58 |  |  |
|  | Pz | |  |  |  |  |  |  |  |
|  |  | eHealth | 21 | 7.2 (2.5) | 9.5 (11.2) | 0.85 | < 0.41 | 3.1 (-3.0, 9.3) | 0.309 |
|  |  | TAU | 19 | 6.9 (3.0) | 7.7 (4.3) | 0.78 | < 0.44 |  |  |
|  | P4 | |  |  |  |  |  |  |  |
|  |  | eHealth | 21 | 6.7 (3.5) | 6.3 (2.4) | -0.62 | < 0.54 | -1.6 (-4.1, 0.9) | 0.195 |
|  |  | TAU | 19 | 8.0 (3.6) | 8.5 (4.8) | 0.49 | < 0.63 |  |  |
|  | O1 | |  |  |  |  |  |  |  |
|  |  | eHealth | 21 | 7.4 (2.0) | 8.7 (5.6) | 1.04 | < 0.31 | -1.3 (-4.0, 3.7) | 0.947 |
|  |  | TAU | 19 | 8.0 (3.3) | 9.7 (5.1) | 1.65 | < 0.12 |  |  |
|  | O2 | |  |  |  |  |  |  |  |
|  |  | eHealth | 21 | 7.6 (2.2) | 6.9 (3.6) | -0.95 | < 0.35 | -1.9 (-5.0, 1.3) | 0.234 |
|  |  | TAU | 19 | 8.5 (4.9) | 9.3 (5.9) | 0.65 | < 0.52 |  |  |
| Note: TAU, treatment as usual. ANCOVA adjusted for age (continuous variables), BMI (continuous variables), IBS subtype (IBS-C, IBS-D, IBS-M, IBS-U), and baseline power percentages (continuous variables). The p value was calculated using Bonferroni correction. The total power was obtained by adding the powers from 2.0 to 29.8 Hz, analyzed by the fast Fourier transform method. Total potency was expressed as the percentage of the total power. The relative power spectra in the alpha (8.0–12.8 Hz) and beta (13.0–29.8 Hz) bands were expressed as the percentages of the total power (delta [2.0–3.8 Hz], theta [4.0–7.8 Hz], alpha [8.0–12.8 Hz], and beta [13.0–29.8 Hz]). | | | | | | | | | |
